# Gene delivery to hypoxic cells in vitro

**DOI:** 10.1054/bjoc.2000.1318

**Published:** 2000-08-16

**Authors:** G U Dachs, C Coralli, S L Hart, G M Tozer

**Affiliations:** Tumour Microcirculation Group, Gray Laboratory Cancer Research Trust, Mount Vernon Hospital, Northwood, HA6 2JR, UK; Molecular Immunology Unit, Institute of Child Health, University College, London, WC1N 1EH, UK

**Keywords:** green fluorescent protein, tumour conditions, anoxia

## Abstract

Hypoxia in solid tumours has been correlated with poor prognosis and resistance to radiation and chemotherapy. Hypoxia is also a strong stimulus for gene expression. We previously proposed a gene therapy approach which exploits the presence of severe hypoxia in tumours for the induction of therapeutic genes. Hypoxic cells are known to have a reduced metabolic rate, transcription and translation. These facts may prevent gene transfer and therefore warranted further investigation. In this paper the feasibility of gene delivery in vitro under tumour conditions was demonstrated. DNA was delivered in vitro using a peptide-mediated non-viral system. Across a range of oxygen tensions and mammalian cell lines (including human tumour and endothelial cells) it was shown that hypoxic cells could be transfected. Transfection efficiencies varied depending on the level of hypoxia, cell characteristics and gene promoters used. An in vitro model of hypoxia/reoxygenation, designed to mimic the variable nature of tumour hypoxia, showed that hypoxic preconditioning and reoxygenation alone did not reduce transfection efficiency significantly; only chronic anoxia reduced transfection. The fact that neither intermediate hypoxia nor intermittent anoxia significantly reduced transfection is promising for future hypoxia-targeted gene therapy strategies. © 2000 Cancer Research Campaign


*British Journal of Cancer* (2000) **83**(5), 662–667
© 2000 Cancer Research Campaign
doi: 10.1054/ bjoc.2000.1318, available online at http://www.idealibrary.com on IDEAL®

### Gene delivery to hypoxic cells in vitro

**GU Dachs[a], C Coralli[a], SL Hart[b] and GM Tozer[a]**

[a]Tumour Microcirculation Group, Gray Laboratory Cancer Research Trust, Mount Vernon Hospital, Northwood, HA6 2JR, UK; [b]Molecular Immunology Unit, Institute of Child Health, University College London, WC1N 1EH, UK

**Summary** Hypoxia in solid tumours has been correlated with poor prognosis and resistance to radiation and chemotherapy. Hypoxia is also a strong stimulus for gene expression. We previously proposed a gene therapy approach which exploits the presence of severe hypoxia in tumours for the induction of therapeutic genes. Hypoxic cells are known to have a reduced metabolic rate, transcription and translation. These facts may prevent gene transfer and therefore warranted further investigation. In this paper the feasibility of gene delivery in vitro under tumour conditions was demonstrated. DNA was delivered in vitro using a peptide-mediated non-viral system. Across a range of oxygen tensions and mammalian cell lines (including human tumour and endothelial cells) it was shown that hypoxic cells could be transfected. Transfection efficiencies varied depending on the level of hypoxia, cell characteristics and gene promoters used. An in vitro model of hypoxia/reoxygenation, designed to mimic the variable nature of tumour hypoxia, showed that hypoxic preconditioning and reoxygenation alone did not reduce transfection efficiency significantly; only chronic anoxia reduced transfection. The fact that neither intermediate hypoxia nor intermittent anoxia significantly reduced transfection is promising for future hypoxia-targeted gene therapy strategies. © 2000 Cancer Research Campaign

**Keywords:** green fluorescent protein; tumour conditions; anoxia

Solid tumours often have insufficient blood supply, resulting in regions with reduced oxygen concentrations (Vaupel et al, 1991). Diffusion limited hypoxia is a characteristic of solid tumours which has been appreciated for many years (Thomlinson and Gray, 1955). However, it is now recognized that hypoxic cells can also arise from perfusion driven changes in oxygen supply, resulting in rapid and reversible changes in oxygenation (Brown, 1979; Chaplin et al, 1987; Kimura et al, 1996). Hypoxic cells are resistant to radiation (Thomlinson and Gray, 1955) and to some chemotherapeutic agents (Teicher et al, 1981; Hoeckel et al, 1999) and are generally perceived as a hindrance to cancer therapy. However, hypoxia is also a tumour characteristic which is potentially exploitable using bioreductive drugs (Workman and Stratford, 1993) or gene therapy (Dachs et al, 1997).

Hypoxia is a strong stimulus for gene expression. Hypoxic gene regulation consists of two main components, namely an oxygen sensitive transcription factor (hypoxia inducible factor-1, HIF-1) and its recognition sequence (hypoxia regulatory element, HRE) (for a recent review see O'Rourke et al, 1997). HIF-1 activity is regulated primarily by low oxygen conditions, but also via specific oncoproteins and tumour suppressors, such as p53 and the von Hippel Lindau protein (for a recent review see Dachs and Tozer, 2000). The HIF-1/HRE system was found to be common to all mammalian cells tested to date (Maxwell et al, 1993) and, recently, the HIF-1α subunit was shown to be overexpressed in the majority of clinical tumour samples tested (Zhong et al, 1999). We have previously proposed a gene therapy approach based on HRE-regulated gene expression which exploits the presence of severe hypoxia in tumours for the induction of therapeutic genes (Dachs et al, 1997). Chimeric promoters containing hypoxia regulated enhancer sequences were shown to regulate the expression of the marker gene CD2 both in tissue culture and in solid tumours in response to hypoxia. In vitro work using the bacterial cytosine deaminase in a gene directed enzyme prodrug therapy approach demonstrated that the hypoxic induction of this gene could render cells sensitive to the prodrug 5-fluorocytosine (Dachs et al, 1997).

The one fundamental question remaining from this work was the feasibility of gene delivery to hypoxic regions of solid tumours. Hypoxia reduces cell proliferation, induces growth arrest and specifically inhibits replicon initiation in early S-phase within 8–24 h in most cells (for reviews see Giaccia, 1996; Wang and Semenza, 1996). Even though some stress response genes (such as the HIF-1 regulated genes) are up-regulated under hypoxia, the majority of cell metabolism is decreased, with a reduction in overall transcription and translation. Protein synthesis rates under hypoxia (<0.01% $O_2$) were reduced to 60% of the aerobic rate (Heacock and Sutherland, 1986). A reduction in RNA and DNA content of hypoxic cultures, as well as a reduction in the de novo synthesis of nucleotides had also been reported (Loeffler, 1992). These effects may prevent gene delivery to hypoxic cells in solid tumours and therefore the effect of hypoxia on transfection efficiency warranted further investigation.

The aim of this study was to analyse in vitro the feasibility of gene uptake under conditions which mimic tumour hypoxia. A non-viral transfection method was chosen for the study of hypoxic cell transfection. This DNA delivery system, which consists of an integrin-targeting peptide, lipofectin and plasmid DNA which combine electrostatically to form a complex, has demonstrated efficient transfection of a range of cell types (Hart et al, 1995, 1998).

*Received 31 January 2000*
*Revised 5 May 2000*
*Accepted 5 May 2000*

*Correspondence to:* GU Dachs

**List of abbreviations**: HIF-1, hypoxia inducible factor 1; CMV, cytomegalovirus; SV40, simian virus 40; $AnO_2$, anoxia; GFP, green fluorescent protein; FACS, fluorescent activated cell sorting

Transfection efficiencies of two commonly used marker genes and viral promoters were recorded in a range of cell lines under different oxygenation conditions. A simple in vitro model of hypoxia/reoxygenation was used to mimic the variable nature of tumour hypoxia.

### MATERIALS AND METHODS

### Cell lines and growth media

The human bladder carcinoma cell line T24 (European Collection of Cell Cultures; Bubenik et al, 1973; Dirks et al, 1999), the immortalized human microvascular endothelial cell line HMEC-1 (Ades et al, 1992), human squamous carcinoma cell line of the head and neck FaDu (Rangan, 1972; American Type Culture Collection), the mouse hepatoma cell line Hepa-1 wild type (clone Hepa-1c1c7) and its derivative Hepa c4 (HIF-1β mutant; Hankinson, 1979) were utilised. Only cells tested negative for mycoplasma were used in the experiments, since it had been shown that mycoplasma interfered with transfection, especially under hypoxia (results not shown). All cells were maintained in Dulbecco's MEM (DMEM, Life Technologies, UK) with 10% fetal calf serum and L-glutamine in a 5% $CO_2$ humidified incubator at 37°C.

Cell proliferation was monitored by cell counting using a haemocytometer and trypan blue (0.2% final concentration, Sigma) to distinguish live and dead cells.

### Hypoxic conditions

Cells were plated 16–24 h prior to the experiment in oxygen impermeable dishes (Permanox, Nunc, UK) at 1–5×10$^5$/dish (4–18×10$^3$/cm$^2$), depending on the growth rate of the cell line. Hypoxic conditions were achieved by placing the dishes (with a thin layer of pre-conditioned anoxic media above the cells) either into sealed containers flushed continuously with humidified 1% or 0.1% $O_2$ (in 5% $CO_2$, balance $N_2$) (BOC Gases, UK), or by placing the dishes in an anaerobic glove cabinet (in an atmosphere of 5% $CO_2$, 5% $H_2$, 90% $N_2$, with palladium catalyst; Don Whitley Scientific Ltd, UK). All hypoxic manipulations were carried out in the anaerobic glove cabinet to minimise the amount of oxygen in the hypoxic experiments by moving the sealed containers into the glove cabinet. The media pH was monitored and found to be the same in air and anoxic conditions (pH 7.5).

### Transfection method

Cells were transfected using integrin-targeting peptides (sequence: $K_{16}$GACRRETAWACG) and lipids as described previously (Hart et al, 1998). Briefly, a mixture was prepared containing lipofectin (Life Technologies, UK), peptide and plasmid DNA at a ratio of 0.75:4:1 by weight in OptiMEM (Life Technologies). The mixture was left at room temperature for 2 h for complex formation, then diluted into OptiMEM. Cells were left to equilibrate for 2 h under hypoxia or anoxia prior to transfection. After washing the cells in phosphate buffered saline (PBS), the mixture was added to the cells to transfect for 5 h (unless specified) at 37°C in air or hypoxia. The number of cells expressing the marker proteins was assessed 24 h later.

### DNA plasmids and product detection

General molecular biology techniques were used for DNA manipulations (Sambrook et al, 1989). The two marker genes, GFP (green fluorescent protein) and CD4, and the two strong viral promoters from cytomegalovirus (CMV) and simian virus (SV40), were chosen for this work since they are commonly used in gene regulation and transfection studies.

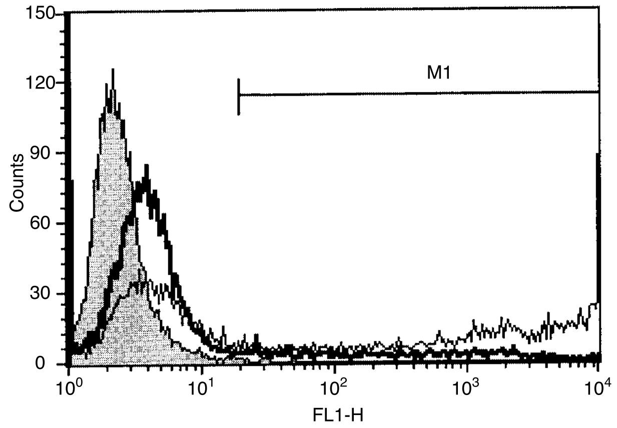

**Figure 1** FACS analysis of transiently transfected human cells. Fluorescence of untransfected control cells (solid histogram), cells transfected in air (–) or under anoxia (**–**) is measured using FACS analysis. A typical scan for HMEC-1 cells following transfection with pEGFP-N1 is shown. Transfection efficiency is defined as the % of cells (of a total of $10^4$ cells analysed) with increased fluorescence above controls (within marker M); < 1% of untransfected cells are contained in M

The commercially available plasmid pEGFP-N1 (Clontech, UK) was employed; it contains the gene encoding the red-shifted, enhanced variant of GFP under the control of the CMV promoter. Direct detection of GFP (excitation 488 nm, emission 507 nm) was done using fluorescent activated cell sorting (FACS, Becton Dickinson, UK) and normalised to untransfected control cells (Figure 1).

The pMACS4.1 (Milteyne Biotec) plasmid encodes a truncated cell surface human CD4 protein under the control of the SV40 promoter. The plasmid pCMV-CD4 was produced as follows: the CD4 encoding gene fragment (digestion of pMACS4.1 with *Pst* 1, followed by Mung Bean nuclease digestion to blunt, followed by *EcoR* 1 digestion) was ligated to the GFP-deleted backbone of pEGFP-N1 (digestion of pEGFP-N1 with *Not* 1, followed by Mung Bean nuclease digestion, followed by *EcoR* 1 digestion). Enzymes and buffers were from Life Technologies. The pMACS4.1 and pCMV-CD4 transfected cells were detected with FITC-labelled anti-human CD4 Ab (Coulter, UK) using FACS and normalised to untransfected, Ab-stained control cells.

### Statistical analysis

Significance tests were carried out on the data groups using analysis of variance (ANOVA) followed by the students t-test for individual pairwise comparisons, with values of $P < 0.05$ considered as significant.

### RESULTS

### Transfection efficiency in air

The initial experiments were done to determine the optimal length of transfection time. Bladder carcinoma T24 cells were exposed to the delivery complex with pEGFP-N1 from 10 s to 7 h in air and

© 2000 Cancer Research Campaign

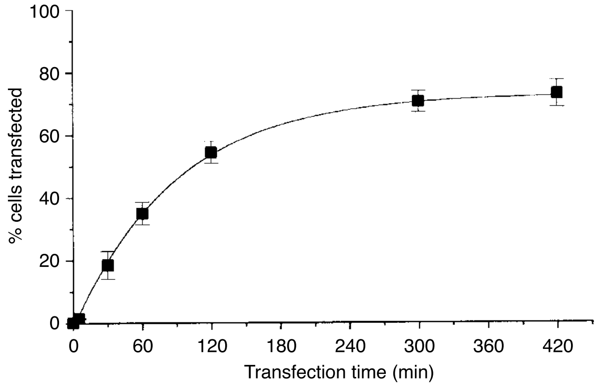

Figure 2 Lengh of transfection time affects percentage of transfected cells. The % of transfected T24 cells increased over time of treatment with a peptide-targeted lipofectin vector containing pEGFP-N1; half maximum transfection was achieved within 1 h and maximal transfection was achieved within 5 h. Means of at least 3 independent experiments, in duplicate, ± standard error (SE) are shown

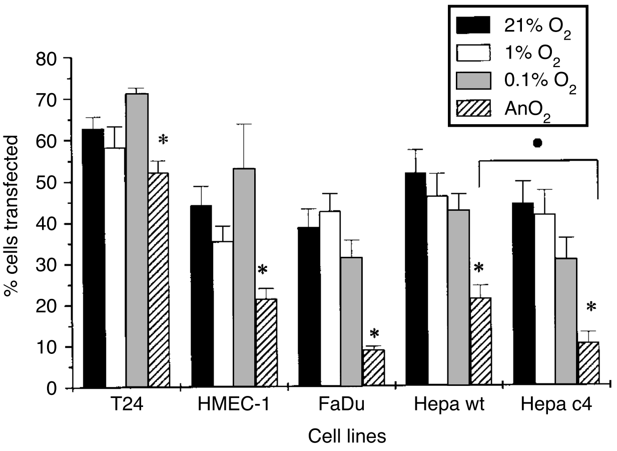

Figure 3 Transfection efficiency in mammalian cell lines with pEGFP-N1 under different oxygenation conditions. Anoxia reduced transfection significantly (* $P < 0.01$ by students t-test) in all five cell lines compared to both air and moderate hypoxia (except for T24 at 1% $O_2$). Hepac 4 cells showed a significantly reduced transfection efficiency compared to Hepa wt under anoxia (* $P<0.05$). Means of at least 3 independent experiments, in duplicate, ±SE are shown analysed for GFP fluorescence 24 h later (Figure 2). Half-maximal transfection was achieved within 1 h. A plateau in transfection efficiency was reached by 5 h transfection time and this transfection period was used in all subsequent experiments.

Transfection efficiency of a panel of mammalian cell lines, including cancer cell lines and endothelial cells, using the peptide-mediated gene delivery was analysed. Between 41 and 69% of cells expressed the stabilized form of GFP 24 h after transfection for 5 h with pEGFP-N1 in air (Figure 3, solid black bars). T24 bladder carcinoma cells showed the highest level of transfection, while FaDu squamous carcinoma cells showed the lowest. Analysis of growth characteristics showed that T24 cells grew faster than FaDu cells (doubling times of about 18 h and 34 h, respectively).

### Hypoxic transfection efficiency: effect of $O_2$ concentration and cell characteristics

The tranfection efficiency of mammalian cells was tested under a range of oxygen concentrations (anoxia, 0.1%, 1% and 21% $O_2$ during transfection with pEGFP-N1 complex and for 24 h afterwards). The exposure to moderate hypoxia (0.1% and 1% $O_2$) did not significantly reduce transfection efficiency in any of the cell lines tested, whereas severe hypoxia (anoxia) reduced it by 28 to 80%, depending on the cell line (Figure 3). In addition, in all cell lines tested it was observed that anoxia reduced the number of highly fluorescent cells ($10^3$-fold above autofluorescence) (Figure 1). These studies show that hypoxia can effect the transfection and/or production of functional proteins in vitro depending on the severity of oxygen deprivation and the cell line.

Anoxia had the least effect on transfection efficiency in the T24 cells and the most effect on FaDu cells. Growth characteristics were unlikely to be the main determining factor here. Both showed a total cessation of growth under anoxia, with anoxia-related cell deaths of 7–13% after 31 h under anoxia (results not shown).

The lack of the hypoxic sensing mechanism appears to be a significant factor of transfection efficiency under anoxia. Comparing the mouse hepatoma cells Hepa wt with Hepa c4, which are mutated for the HIF-1 transcription factor, transfection tended to be slightly less efficient for the mutant cell line (Figure 3). A statistical analysis (two population students t-test) showed this to be significant under anoxic conditions ($P < 0.05$).

### Hypoxic transfection efficiency: effect of transient anoxia

A system of changing oxygenation aimed at mimicking those transient hypoxic conditions found in solid tumours (Brizel et al, 1995; Chaplin and Hill, 1995) was utilized to further test the effect of tumour conditions on gene transfer. Reoxygenation of T24 cells for different lengths of time (0–24 h) after anoxic transfection (2 h + 5 h anoxia) showed the same transfection efficiency as aerobic controls (Figure 4A). The effect of anoxic pre-conditioning, anoxic transfection or anoxic incubation after transfection on gene transfer was analysed (Figure 4B). T24 cells were incubated in air (o) or anoxia (a) prior to transfection (for 24 h), during transfection (for 5 h) and after transfection (for 24 h). Gene transfer under long-term, chronic anoxia (aaa) differed significantly ($P < 0.01$) from transfer under aerobic and transient anoxia (ooo, oao and oaa) (Figure 4B). Transfection efficiency was similar in all other treatment combinations.

### Hypoxic transfection efficiency: effect of gene product and promoter

The GFP chromophore is formed by a cyclization reaction and an oxidation step that requires molecular oxygen (Cubitt et al, 1995). It is also known that some gene promoters are regulated by oxygenation. The effect of the gene product and the promoter on hypoxic transfection efficiency in T24 cells was therefore tested. Two plasmids were utilized; both encoding a truncated version of the surface protein CD4, either under the control of the SV40 (pMACS4.1) or the CMV promoter (pCMV-CD4), and compared them to the pEGFP-N1 plasmid (CMV-controlled GFP). A higher percentage of cells expressed GFP following transfection than CD4 under any given oxygenation condition ($P < 0.05$, with the exception of pMACS4.1 under 1% $O_2$, Figure 5). Oxygenation had no effect on transfection efficiency in cells transfected with either

 © 2000 Cancer Research Campaign

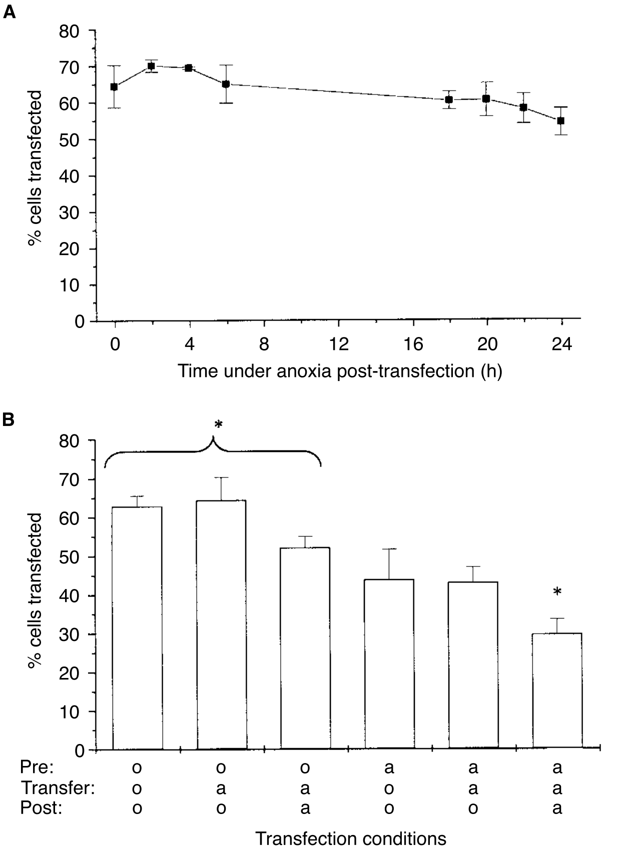

**Figure 4** Transfection efficiency of T24 cells under changing oxygenation conditions. (**A**) Effect of reoxygenation on transfection efficiency. Transfection conditions: anoxic transfection and 0–24 h post-transfection anoxia. (**B**) Effect of pre- or post-anoxia treatment on transfection efficiency. Transfection conditions: 24 h pre-transfection, 5 h transfection, 24 h post-transfection; O = incubation in air, a = incubation under anoxia. Transfection under chronic anoxia (aaa) differs significantly from normoxia and intermittent anoxia (ooo, oao, oaa); * $P < 0.01$ by students t-test. Means of at least 3 independent experiments, in duplicate, ± SE are shown

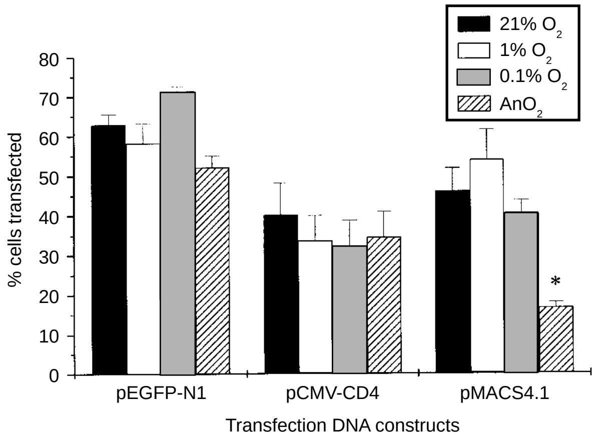

**Figure 5** Effect of gene product or promoter on transfection in T24 cells under a range of oxygenation conditions. DNA constructs used: pEGFP-N1 (CMV promoter – GFP), pCMV-CD4 (CMV promoter – truncated CD4), pMACS4.1 (SV40 promoter – truncated CD4). Only transfection efficiencies with pMACS4.1 under anoxia are significantly reduced compared to transfection with any other construct under any conditions; * $P < 0.01$ by students t-test. Means of at least 3 independent experiments, in duplicate, ± SE are shown

CMV promoter-controlled GFP or CD4 DNA constructs. However, T24 cells transfected with the DNA construct encoding CD4 under SV40 promoter-control showed a significant reduction in transfection efficiency under anoxia compared to the aerobic control (by 64%) and mild hypoxia (Figure 5). The reduced anoxic transfection efficiency observed in cells transfected with pMACS4.1 appears therefore to depend mostly on the SV40 promoter rather than the gene product in this cell line.

### DISCUSSION

Gene delivery remains the main stumbling block in the gene therapy of cancer. Both viral and non-viral delivery methods are under intense investigation, with non-viral means holding the promise of safety and specificity. The introduction of foreign gene products into human cells consists of a sequence of discrete steps. First the vector must bind to the cell surface, then enter the cell by an endocytic or phagocytic process. Efficient escape from the vesicle is necessary to evade enzymatic degradation in the acidified environment of the maturing endosome. Finally the DNA must enter the nucleus either by penetrating the envelope through the nuclear pores or during mitosis when the envelope breaks down. The persistence of gene expression can be limited by shut-off of promoters, restrictive methylation or one or more of a number of post-transcriptional events. For most cancer gene therapy strategies, however, such as suicide gene therapy or immunotherapy, transient gene expression should be sufficient.

The DNA delivery system used in this investigation consists of integrin-targeting peptides, lipofectin and plasmid DNA (Hart et al, 1995, 1998). The peptide portion of the delivery system contains a 16-lysine DNA-binding domain and one of a number of integrin-binding domains. The cationic charge properties of the complex, derived from the peptide, contribute to non-specific cell binding and transfection properties (Hart et al, 1998). The lipid component acts as an adjuvant to transfection by mediating endosomal escape of the plasmid component.

Integrins are membrane glycoproteins important for attachment of cells to the extracellular matrix, cell–cell interactions, migration and signalling events (for a review see Hynes, 1992). They are also exploited for cell binding and entry by a number of bacterial and viral pathogens. Therefore, the advantage of this approach is that it mimics cell entry of pathogens via integrin receptors. Integrins on cancer cells play important roles in tumour growth, angiogenesis and metastasis. Integrin expression patterns in tumour cells often differ from their normal counterparts, offering windows of opportunity for tumour-specific transfection.

Using this gene transfer system a plateau of maximal transfection efficiency of about 70% was reached within 5 h. In T24 cells a residual 20–30% of cells remained refractive to transfection, even after 24 h of transfection (results not shown), which may be a cell cycle effect. The effect of cell proliferation on transfection efficiency remains unclear. While some of our preliminary observations showed no effect of cell cycle on transfection (by DNA staining and FACS analysis), others showed that non-cycling aerobic cells (using more confluent cultures) were more difficult to transfect (unpublished data) and cells with increased proliferation transfected more efficiently (comparing T24 and FaDu cells in Figure 3).

The tumour microenvironment is characterized by high interstitial fluid pressure, low extracellular pH, temporally and spatially

© 2000 Cancer Research Campaign heterogeneous blood flow and hypoxia (Helmlinger et al, 1997) which are all a challenge for gene delivery. In this paper we focused on hypoxia as a potential problem in gene tranfection as it is the chemo- and radio-resistant hypoxic cells that we wish to target for our gene therapy strategy. In addition, although a set of stress response genes are specifically induced by hypoxia, tumour hypoxia is known to reduce overall transcription and protein production which could reduce transfection efficiency (Heacock and Sutherland, 1986; for a recent review see Dachs and Chaplin, 1998). The effect of hypoxia on gene delivery therefore required careful analysis prior to the further development of hypoxia-targeted gene therapy.

Hypoxic transfection efficiencies varied between the cell lines tested. Although transfection, using this non-viral gene delivery system, has previously been shown to be largely integrin mediated (Hart et al, 1998), no integrin specificity could be demonstrated in this study and transfer was believed to be mostly charge mediated (own observations). Integrin expression, which is modulated by hypoxia (anoxia decreased and 1% $O_2$ increased integrins $\alpha V\beta 3$ and $\alpha 5\beta 1$ slightly in T24 and HMEC-1 cells; results not shown), is therefore not a factor in determining the effectiveness of transfection under hypoxia in this system. On the other hand, the HIF-1 hypoxia response system appeared to be one significant, although minor, factor in determining anoxic transfection efficiencies (comparing Hepa wt and Hepa c4 in Figure 3).

Anoxia (0 mmHg) has been measured in solid tumours (Hoeckel et al, 1991; Vaupel et al, 1991), but it is currently unclear whether that subpopulation of cells contributes to the outcome of cancer treatment, as they may be completely necrotic. Cells at oxygenation conditions below 2.5 mmHg (roughly 0.3% $O_2$) are known to be significantly more resistant to the effects of ionising radiation than aerated cells. The anoxic conditions achieved using an anaerobic glove compartment with palladium catalyst are more stringent than gassing with a mixture of 95% $N_2$, 5% $CO_2$ (own observations, also Dachs et al, 1997). The chronic anoxia used in this paper was chosen as an extreme case and showed that even under these severe conditions some level of transfection was possible. The findings that radio-biologically relevant hypoxia (0.1% $O_2$) did not reduce gene transfer is encouraging for future gene therapy strategies. Furthermore, conditioning cells with anoxia prior to or after transfections had little effect on transfection efficiency, which indicates that transient hypoxia in tumours is unlikely to reduce gene transfer.

An additional challenge to gene transfer, which is the ability of the delivery vehicle to penetrate through multicell layers, requires future analysis. This challenge may be a problem if the main target for gene therapy is the population of chronic hypoxic tumour cells at a distance from the nearest blood vessel. However, a more likely target may be the transient hypoxic population of cells in the tumour, especially endothelial cells lining the blood vessels. Tumour microvessels have been shown to be subject to acute periods of hypoxia in vivo (Kimura et al, 1996). Targeting endothelial cells for hypoxia-selective gene therapy has several advantages: endothelial cells are systemically accessible, are not transformed and support large numbers of tumour cells. Data presented in this paper show that hypoxic human endothelial cells can be transfected.

When analysing transfection efficiencies the choice of marker gene is important. In this paper all cells transiently transfected with GFP under anoxic conditions showed a reduction in highly fluorescent cells (thousand-fold above autofluorescence) when compared to those transfected in air or mild hypoxia. It is known that the marker protein GFP requires molecular oxygen to fold correctly and fluoresce (Cubitt et al, 1995). Analysis of stable GFP transfectants had shown that anoxia reduced GFP fluorescence, whereas less severe hypoxia (including gassing with 95% $N_2$, 5% $CO_2$) did not significantly alter GFP fluorescence (Coralli et al, manuscript in preparation). In an attempt to avoid hypoxic effects on the detection of the marker gene GFP, a second marker gene, encoding the truncated CD4, was used. Oxygenation conditions made no significant difference to transfection of either gene product under CMV promoter control (Figure 5). However, a difference in transfection efficiency under anoxia was observed when the marker genes were controlled by different promoters. A significant reduction in anoxic transfection efficiency was observed in T24 cells transfected with the SV40-controlled marker gene and not with CMV-controlled marker genes. Both the CMV and SV40 promoters originate from viruses and are used widely as strong constitutive regulators of gene expression. From the results presented here it appears that expression controlled by the SV40 promoter is more oxygen sensitive then that controlled by the CMV promoter.

In conclusion, transfection efficiencies were shown to be significantly effected by cell line characteristics, the gene promoter and severe tumour conditions such as anoxia. The fact that neither intermediate hypoxia nor intermittent anoxia had significant detrimental effects on gene transfer is promising for future hypoxia-targeted gene therapy strategies.

### ACKNOWLEDGEMENTS

We would like to thank Dr Azza Khalil for helping with the statistical analysis and Dr Maja Cemazar for critical reading of the manuscript. We gratefully acknowledge the financial support from the Cancer Research Campaign [CRC], grant number SP2292/0102.

### REFERENCES

Ades EW, Candal FJ, Swerlick RA, George VG, Summers S, Bosse DC and Lawley TJ (1992) HMEC-1: Establishment of an immortalised human microvascular endothelial cell line. *J Invest Dermatol* **99**: 683–690

Brizel DM, Rosner GL, Prosnitz LR and Dewhirst MW (1995) Patterns and variability of tumour oxygen in human soft-tissue sarcomas, cervical carcinomas and lymph-node metastases. *Int J Radiat Onc Biol Phys* **32**: 1121–1125

Brown JM (1979) Evidence for acutely hypoxic cells in mouse tumours, and a possible mechanisms of reoxygenation. *Br J Radiol* **52**: 650–656

Bubenik J, Baresova M, Viklicky V, Jakoubkova J, Sainerova H and Donner J (1973) Established cell line of urinary bladder carcinoma (T24) containing tumour-specific antigen. *Int J Cancer* **11**: 765–773

Chaplin DJ and Hill SA (1995) Temporal heterogeneity in microregional erythrocyte flux in experimental solid tumours. *Br J Cancer* **71**: 1210–1213

Chaplin DJ, Olive PL and Durand RE (1987) Intermittent blood flow in a murine tumour: radiobiological effects. *Cancer Res* **47**: 597–601

Cubitt AB, Heim R, Adams SR, Boyd AE, Gross LA and Tsien RY (1995) Understanding, improving and using green fluorescent proteins. *Trends Biochem Sci* **20**: 448–455

Dachs GU and Chaplin DJ (1998) Microenvironmental control of gene expression: Implication for tumour angiogenesis, progression and metastasis. *Seminars in Radiation Oncology* **8**: 208–216

Dachs GU and Tozer GM (2000) Hypoxia modulated gene expression: angiogenesis, metastasis and therapeutic exploitation. *Eur J Cancer*, in press

Dachs GU, Patterson AV, Firth JD, Ratcliffe PJ, Townsend KMS, Stratford IJ and Harris AL (1997) Targeting gene expression to hypoxic tumour cells. *Nature Med* **3**: 515–520

© 2000 Cancer Research Campaign

Dirks WG, MacLeod RAF, Drexler HG (1999) ECV304 (endothelial) is really T24 (bladder carcinoma): cell line cross-contamination at source. *In Vitro Cell Develop Biol* **35**: 558–559

Giaccia AJ (1996) Hypoxic stress proteins: Survival of the fittest. *Sem Rad Oncol* **6**: 46–58

Hankinson O (1979) Single step selection of clones of a mouse hepatoma line deficient in aryl hydrocarbon hydroxylase. *Proc Natl Acad Sci* USA **76**: 373–376

Hart SL, Harbottle RP, Cooper R, Miller A, Williamson R and Coutelle C (1995) Gene delivery and expression mediated by an integrin-binding peptide. *Gene Ther* **2**: 552–554

Hart SL, Arancibia-Carcamo CV, Wolfert MA, Mailhos C, O'Reilly NJ, Ali RR, Coutelle C, George AJT, Harbottle RP, Knight AM, Larkin DFP, Levinsky RJ, Seymour LW, Thrasher AJ and Kinnon C (1998) Lipid-mediated enhancement of transfection by a nonviral integrin-targeting vector. *Human Gene Ther* **9**: 575–585

Heacock CS and Sutherland RM (1986) Induction characteristics of oxygen regulated proteins. *Int J Radiation Oncology Biol Phys* **12**: 1287–1290

Helmlinger G, Yuan F, Dellian M and Jain RK (1997) Interstitial pH and $pO_2$ gradients in solid tumours in vivo: high resolution measurements reveal a lack of correlation. *Nat Med* **3**: 177–182

Hoeckel M, Schlenger K, Knoop C and Vaupel P (1991) Oxygenation of carcinomas of the uterine cervix: Evaluation of computerised $O_2$ tension measurements. *Cancer Res* **51**: 6098–6102

Hoeckel M, Schlenger K, Hoeckel S and Vaupel P (1999) Hypoxic cervical cancers with low apoptotic index are highly aggressive. *Cancer Res* **59**: 4525–4528

Hynes RO (1992) Integrins: versatility, modulation, and signalling in cell adhesion. *Cell* **69**: 11–25

Kimura H, Braun RD, Edgardo TO, Ong ET, Hsu R, Secomb TW, Papahadjopoulos D, Hong KL and Dewhirst MW (1996) Fluctuations in red cell flux in tumour microvessels can lead to transient hypoxia and reoxygenation in tumour parenchyma. *Cancer Res* **56**: 5522–5528

Loeffler M (1992) A cytokinetic approach to determine the range of $O_2$-dependence of pyrimidine(deoxy)nucleotide biosynthesis relevant for cell proliferation. *Cell Prolif* **25**: 169–179

Maxwell PH, Pugh CW and Ratcliffe PJ (1993) Inducible operation of the erythropoietin 3′ enhancer in multiple cell lines: evidence for a widespread oxygen sensing mechanism. *Proc Natl Acad Sci* USA **90**: 2423–2427

O'Rourke JF, Dachs GU, Gleadle JM, Maxwell PH, Pugh CW, Stratford IJ, Wood SM and Ratcliffe PJ (1997) Hypoxia response elements. *Oncology Res* **9**: 327–332

Rangan SR (1972) A new human cell line (FaDu) from a hypopharyngeal carcinoma. *Cancer* **29**: 117–121

Sambrook J, Fritsch EF and Maniatis T (1989) *Molecular cloning. A laboratory manual*. Cold Spring Harbor Laboratory Press: Cold Spring Harbor

Teicher BA, Lazo JS and Sartorelli AC (1981) Classification of antineoplastic agents by their selective toxicities towards oxygenated and hypoxic tumour cells. *Cancer Res* **41**: 73–81

Thomlinson RH and Gray LH (1955) The histological structure of some human lung cancers and the possible implication for radiotherapy. *Br J Cancer* **9**: 539–549

Vaupel P, Schlenger K, Knoop C and Hoeckel M (1991) Oxygenation of human tumours: Evaluation of tissue oxygen distribution in breast cancers by computerised $O_2$ tension measurements. *Cancer Res* **51**: 3316–3322

Wang GL and Semenza GL (1996) Oxygen sensing and response to hypoxia by mammalian cells. *Redox Report* **2**: 89–96

Workman P and Stratford IJ (1993) The experimental development of bioreductive drugs and their role in cancer therapy. *Cancer Metast Rev* **12**: 73–82

Zhong H, DeMarzo AM, Laughner E, Lim M, Hilton DA, Zagzag D, Buechler P, Isaacs WB, Semenza GL and Simons JW (1999) Overexpression of hypoxia-inducible factor 1α in common human cancers and their metastasis. *Cancer Res* **59**: 5830–5835

© 2000 Cancer Research Campaign
